# Editorial

**Published:** 1900-03-15

**Authors:** 


### EDITORIAL.

A VOTE is being taken by postal card on a proposition to change the time of the meeting of the National Dental Association from June 26th to either July 3rd or 10th, so that those who contemplate attending the Dental Congress at Paris in August may not be compelled to make two trips to the East. We wish we could also have a chance to vote for a more desirable place for the meeting. For those who contemplate making a tour of Europe in connection with the trip to Paris, it would seem that June 26th would be a much better date than a later one; but as many who go will not stop longer than for a brief trip, the later date is probably more desirable. The Transportation Committee and the Executive Committee at Paris are doing everything in their power to make the visit one of profit, convenience, and at moderate cost. It is hoped there may be a large American delegation.

NATIONAL legislation of interest to the profession seems to be making hopeful progress. The bill to establish dental surgeons in the Army and Navy seems likely to become a law, and already candidates for the various places are much in evidence. Just how a dentist with any practice can see anything attractive in these places, is one of the curious features of this agitation. The salary certainly can not be an object, and what is there in the life or the future prospect that invites? It must be the intense patriotic spirit of devotion to our noble profession and glorious country. If so, we are glad we are not to be found wanting in generous loyalty to both in this emergency.

A SOMEWHAT more selfish legislative enactment, yet none the less important, is the effort to modify existent patent laws in regard to patenting processes, especially such as there is great question as the priority of claimants, and which are of such great importance in relieving human suffering and preserving the health and comfort of the people. Dr. Ottolengui is pushing this bill in Congress, and he asks—and must have, if he is to succeed—the endorsement and assistance of the profession. Write to your Congressman and call his attention to this bill and ask his support. He will inform you of his attitude. If it is favorable, send the letter to Dr. Ottolengui, 115 Madison Ave., New York, who will make good use of it in securing a hearing for the bill in Congress. It is a worthy measure, and all individuals or societies are urged to co-operate for its enactment as a law to rid the profession of these notorious patent sharks.

